# Analgesic and Anxiolytic Effects of Gastrodin and Its Influences on Ferroptosis and Jejunal Microbiota in Complete Freund’s Adjuvant-Injected Mice

**DOI:** 10.3389/fmicb.2022.841662

**Published:** 2022-04-19

**Authors:** Xin Chen, Jinyue Wang, Zhixian He, Xin Liu, Huawei Liu, Xing Wang

**Affiliations:** ^1^College of Medicine, Southwest Jiaotong University, Chengdu, China; ^2^Department of Laboratory Medicine, The Affiliated Hospital of Southwest Jiaotong University, The Third People’s Hospital of Chengdu, Chengdu, China

**Keywords:** gastrodin, analgesic, anxiolytic, ferroptosis, microbiota

## Abstract

This study investigated the effects of gastrodin (GAS) on analgesic, anxiolytic, ferroptosis, and jejunal microbiota in chronic inflammatory pain mice. The chronic inflammatory pain model of C57BL/6J mice was established by hindpaw injection of complete Freund’s adjuvant (CFA). After GAS treatment, thermal hyperalgesia test, mechanical allodynia test, elevated plus-maze (EPMT), and open-field test (OFT) were performed to assess the behavioral changes of pain and anxiety. mRNAs of FTHI, GPX4, HO-1, and PTGS2 and jejunal microbiota were measured by qPCR. In CFA-injected C57BL/6 mice, we found that the mechanical and thermal pain threshold were increased with treatment of GAS. In EPMT, the number of entries in open arms and retention times of open arms were increased by GAS. In the OFT, the time spent in the central area was also increased. Furthermore, GAS enhanced mRNA expressions of FTHI, GPX4, and HO-1 but decreased the expression of PTGS2 in a dose-dependent manner. GAS is effective in the treatment of mice chronic inflammatory pain and anxiety-like behaviors. It may be exhibits potential neuroprotective effects through inhibition of ferroptosis independently of the intestinal microbiota.

## Introduction

Ferroptosis is a unique iron-dependent form of regulated cell death (Dixon et al., 2012). The accumulation of lipid peroxidation products and lethal reactive oxygen species (ROS) is the main characteristic of ferroptosis (Xie et al., 2016). Ferroptosis, as a way to promote cell death, may be implicated in the occurrence and development of many diseases. Studies have shown the importance of ferroptosis in many diseases of the central nervous system, such as Alzheimer’s disease (AD), Parkinson’s disease (PD), Huntington’s disease (HD), amyotrophic lateral sclerosis (ALS), and traumatic brain injury (TBI; Wu et al., 2018).

**Figure 1:**
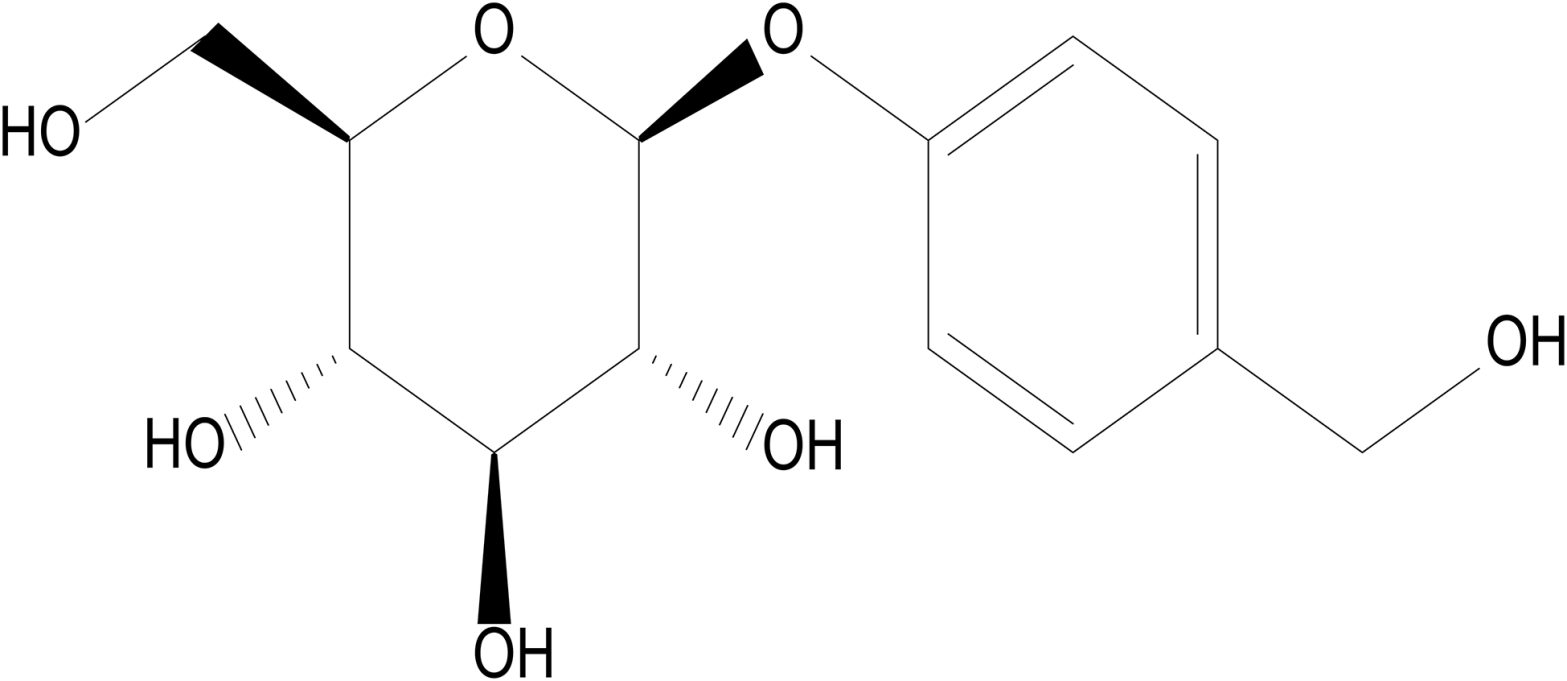
The chemical structural formula of gastrodin (GAS).

*Gastrodiae Rhizoma* (Tianma), a notable Chinese herb, is dry tubers of *Gastrodia elata Blume* which belongs to Orchidaceae. *Gastrodiae Rhizoma* is considered a top-grade medicine described to treat the hypertension of liver-yang hyperactivity in the tradition Chinese medicine. Studies have shown that the application of *gastrodia elatahas* biological activities of anticonvulsion, antioxidation, neuroprotection, anti-denguevirus, anti-cardio-cerebral-vascular diseases, and anti-inflammation (Ahn et al., 2007; Han et al., 2014; Zhan et al., 2016). The major active component and material basis of *Gastrodia elata* are Gastrodin (GAS). GAS, a chemical compound that known as 4-hydroxybenzyl alcohol-4O-β-D-glucopyranoside, is isolated from the rhizome of *Gastrodia elata*. Furthermore, the molecular formula of GAS is C_13_H_18_O_7_, and its chemical structural formula is shown in Figure 1. GAS has numerous pharmacological activities including analgesic (Guo et al., 2013), antidepressant (Chen and Sheen, 2011), anxiolytic (Peng et al., 2013), anti-inflammatory (Kim et al., 2012), antiobesity (Park et al., 2011), and memory and retrieval improvements (Wu et al., 1996; Hsieh et al., 1997). Among them, analgesic, antioxidant, anti-inflammatory, and neuroprotective effects are the main research hotspots in recent years. Recent findings suggest that GAS exerts a protective effect on primary neural progenitor cells (NPCs) by resisting amyloidβ (Aβ; 1–42)-induced neurotoxicity (Li and Qian, 2016). In the meantime, GAS increased the expression of HO-1, Nrf2, and GPX4 protein in Rat Glioma Cell Line C6, which protected Rat Glioma Cell Line C6 from ferroptosis induced by H_2_O_2_ (Jiang et al., 2020). In recent years, several neuroprotective mechanisms of GAS have been fund. However, the study regarding to the effects of Gas on ferroptosis is rare. GAS was also reported to display powerful anti-inflammation properties. Based on the above research progress and analysis, it is speculated that GAS might be a potential therapeutic for the inhibition of ferroptosis. This study was designed to explore the analgesic, anti-inflammatory, and anxiolytic effects of GAS. We also examined whether GAS can exhibit neuroprotective effect through inhibition of ferroptosis, as well as its relation with intestinal microbiota.

## Materials and Methods

### Materials

Gastrodin was purchased from Nanjing Baide Biotechnology Co., Ltd. (>99% purity, Nanjing, China). Complete Freund’s adjuvant (CFA) and Von Frey filaments were purchased from Sigma (St. Louis, MO). Elevated Plus-Maze Video Tracking System was purchased from Shanghai Xinruan Information Technology Co., Ltd. (Shanghai, China). YLS-6A Intelligent hot plate was purchased from Jinan Yiyan Technology Development Co., Ltd. (Shandong, China). ABI7500 Real-Time PCR Detection Systems were purchased from Bio-Rad (Hercules, California). AxyPrep™ Multisource Total RNA was purchased from AXYGEN (Silicon Valley, California). SYBR Green qPCR Mix (2×) was purchased from Beyotime Biotechnology (Shanghai, China). D7260 Prime Script™ RT Reagent Kit was purchased from TaKaRa (Liaoning, China); RR037A primer was purchased from Sangon Biotech (Shanghai, China).

### Animals and Grouping

Male C57BL/6J mice (aged 8 weeks, weighing 21–25 g) were purchased from Chengdu Dashuo Laboratory Animal. Animals were housed in groups of six mice with a temperature (20 ± 2°C), humidity (55 ± 15%), and lighting (12 h light/dark cycle, lights on at 7:00 AM). All animals must adapt to conditions for at least 7 days after they arrived. Food and water were freely available. All experimental procedures were approved by the Animal Ethics Committee of Southwest Jiaotong University and were conducted in accordance with the university’s animal experiment guidelines.

The rats were randomly divided into four groups of six individuals each as follows: Blank group [physiological saline (SAL)-treated group, *n* = 6], model group (The CFA-induced plus SAL-treated group, *n* = 6), the CFA-induced plus 100 mg/kg GAS-treated group (CFA + GAS 100 group, *n* = 6), and the CFA-induced plus 200 mg/kg GAS-treated group (CFA + GAS 200 group, *n* = 6).

### Experimental Designs and GAS Treatment

A total of 10 μl CFA (50%) was injected intraplantar subcutaneously into the left hindpaws of mice to established chronic peripheral inflammatory pain. In the control group, the same volume of SAL was injected into the hindpaws of mice. GAS was dissolved in saline before use. The mice were intraperitoneally injected with GAS (100 and 200 mg/kg) after CFA insult GAS or saline was used repeatedly in mice once a day for 2 weeks.

### Mechanical Allodynia

Mechanical allodynia was assessed with a set of von Frey filaments on day 1, 4, 7, and 14. Mice were placed on a wire mesh covered with organic glass and acclimated to the environment at least 30 min prior to test. Start with 0.4 mN (#2.44) filament and stimulate the center of left hindpaw until filament bending for 3 s, and the mice have reactions like licking foot or foot lifting.

### Thermal Hyperalgesia

After 14 days of administration, the temperature of the hot plate was set to 55°C. The left hindpaw of mice was placed on the hot plate, and time was recorded when the mice had reactions like foot lifting.

### Elevated Plus-Maze Test

Mice were placed in the central zone of the maze facing the closed arm, and the time was recorded for 5 min. Outcome measures: the number of entries in open arms, retention times of open arms, the number of entries in closed arms, and retention times of closed arms. The number of entries in open arms and retention times of open arms were negatively correlated with anxiety in mice.

### Open-Field Test

Mice were placed in the center of the box, and the time of mice entering the central area was videotaped. The observation time is 5 min.

### Intestinal Histomorphology

Specimens of cross-sections of jejunum were embedded in paraffin wax and cut into 5 μm thick histological sections for hematoxylin and eosin staining. An image processing and analysis system were used to measure tissue sections under a microscope. The villus height, crypt depth, and the ration of villus height to crypt depth (VC) of the jejunum were measured by Image-Pro Plus 6.0.

### Real-Time Quantitative PCR

The total RNA was extracted from the ACC and the spinalcord of the rat lumbosacral enlargement (L4-5) using TRIZOL reagent (TaKaRa, Dalian, China). D7260 Prime Script™ RT Reagent Kit performed reverse transcription for the synthesis of cDNA. Reverse transcription was the performed *via* Real-Time PCR System in a 20 μl reaction mixture and while following the manufacturer’s instructions. SYBR Green qPCR Mix (2×) was used for QRT-PCR. The primers utilized here are shown in (Table 1).

**Table 1:** Specific gene primes sequences.

Gene name	Forward prime (5'–3')	Reverse prime (5'–3')	Accession number
HO-1	AGACACCGCTCCTCCAGT	TCAGGTATCTCCCTCCATT	NM_010442
FTH1	GCAGGATATAAAGAAACCAGA	TCTCAATGAAGTCACATAAGT	NM_010239
GPX4	GTCTGGCAGGCACCATGT	GTGACGATGCACACGAAACC	NM_008162
PTGS2	TGGAGGCGAAGTGGGTTTTA	GAGTGGGAGGCACTTGCATT	NM_011198
GAPDH	GCAGAATTCCTGGCCAAGGTCATCCATGAC	GCAGGTACCGGGGCCATCCACAGTCTTCTG	NM_001289726

### Jejunal Microbiota Analysis

Bacterial DNA was extracted from jejunal digesta using the QIAamp DNA Stool Kit (Qiagen, Hilden, Germany) according to the manufacturer’s instructions. Bacterial DNA extracted from the jejunal digesta was used for gene sequence amplification by quantitative PCR using the primers specified in Table 2. Primer specificity was assessed on the basis of the 16S rRNA gene sequence. The reaction conditions for quantitative PCR were as follows: 50°C for 2 min, 95°C for 5 min and 40 cycles of denaturation at 94°C for 20 s, primer annealing at a species-specific temperature for 30 s, and primer extension at 60°C for 1 min.

**Table 2:** Primes for real-time PCR of bacteria.

Item	Primer sequence (5'–3')	Amplicon length (bp)
Bacteroidetes	Forward: GGARCATGTGGTTTAATTCGATGAT	126
	Reverse: AGCTGACGACAACCATGCAG	
Firmicutes	Forward: GGAGYATGTGGTTTAATTCGAAGCA	126
	Reverse: AGCTGACGACAACCATGCAC	
*Lactobacillus*	Forward: AGCAGTAGGGAATCTTCCA	345
	Reverse: ATTCCACCGCTACACATG	

### Statistical Analysis

All results are presented as mean ± standard deviation (SD) and were analyzed using SPSS (Version 13.0, Chicago, United States). A *p* value <0.05 was considered to be statistically significant.

## Results

### Effects of GAS on CFA-Induced Mechanical and Thermal Hypersensitivity

**Figure 2:**
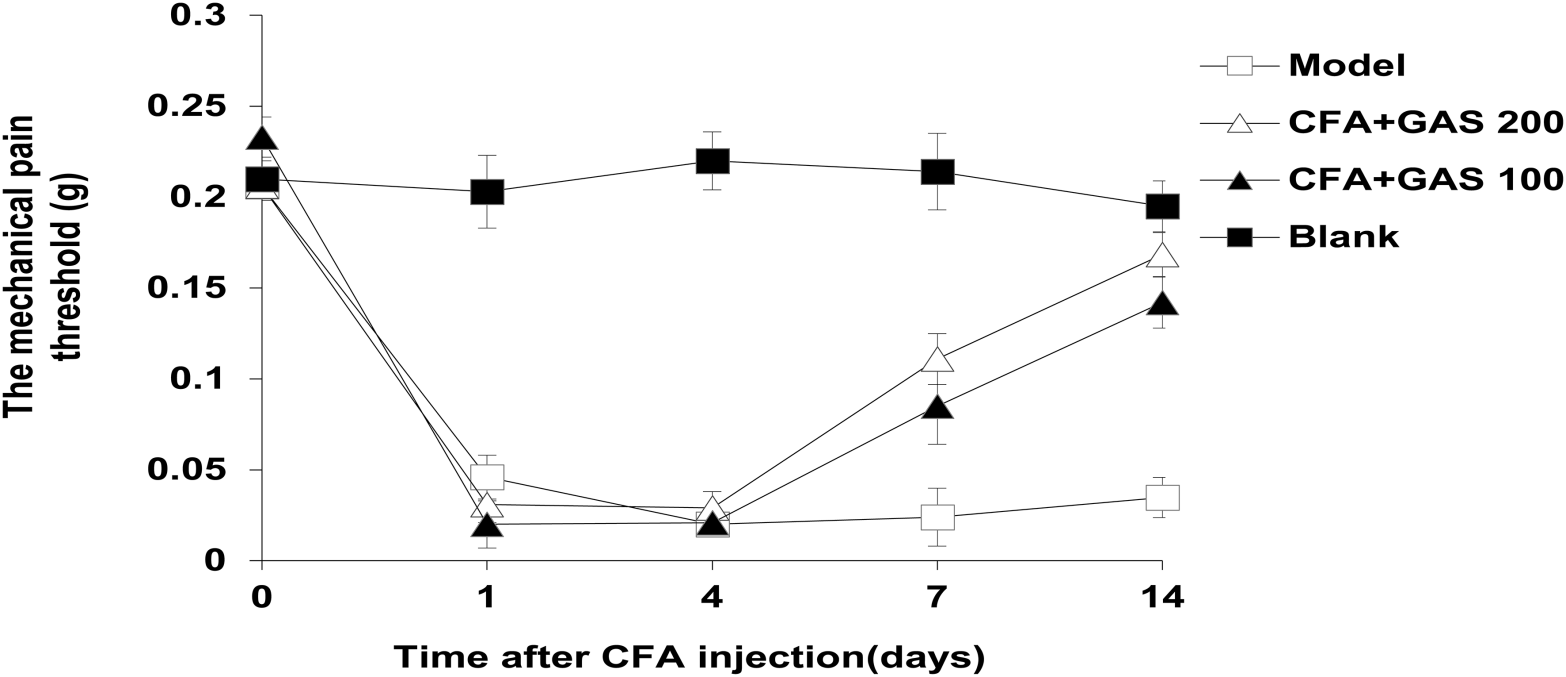
Changes of the paw withdrawal threshold after complete Freund’s adjuvant (CFA) injection.

**Figure 3:**
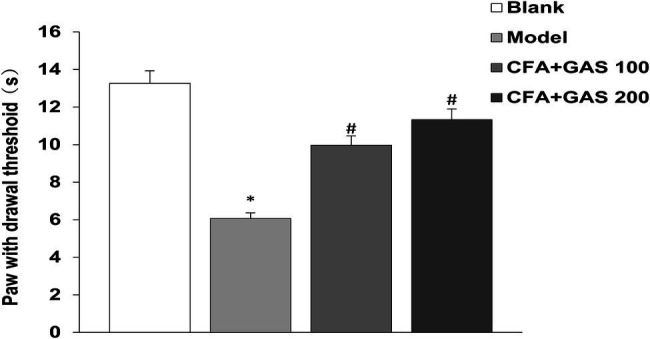
The paw withdrawal latency of mice(s) (^*^p < 0.05 compared with blank group; ^#^*p* < 0.05 compared with model group).

After CFA was injected into mice, mechanical thresholds were determined on day 1, 4, 7, and 14. As shown in Figure 2, on the first day after CFA injection, the mechanical pain threshold of the model group was significantly lower than blank group, and the left hindpaw of mice was obviously swollen, indicating that the chronic inflammatory pain model was successfully established. The paw withdrawal threshold of CFA-injected mice significantly decreased after CFA injection for 1–4 days. Meanwhile, the administration of GAS (100 and 200 mg/kg) increased the paw withdrawal threshold in CFA-injected mice. GAS also attenuated thermal hyperalgesia in CFA-injected mice (Figure 3). Moreover, GAS dose-dependently increased the mechanical and thermal pain threshold in mice.

### Effects of GAS on CFA-Induced Anxiety-Like Behavior

**Figure 4:**
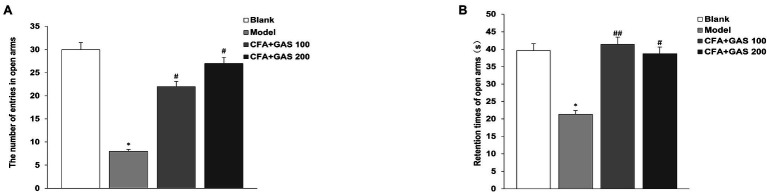
Effect of GAS on results of Morris water maze test. **(A)** The number of entries in open arms. **(B)** Retention times of open arms (^*^p < 0.05 compared with blank group; ^##^*p* < 0.01 and ^#^*p* < 0.05 compared with model group).

**Figure 5:**
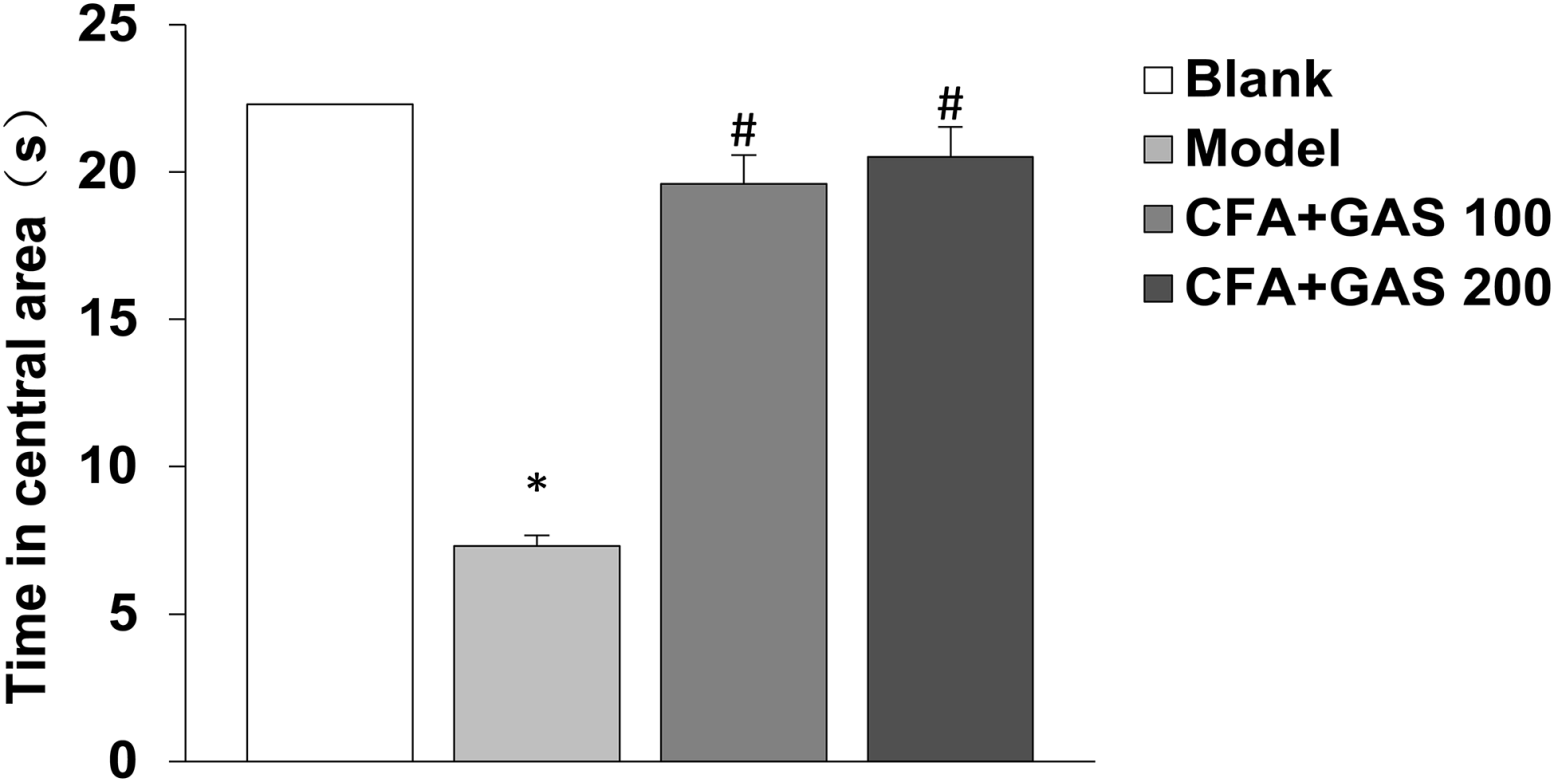
Effect of GAS on results of OFT (^*^p < 0.05 compared with blank group; ^#^*p* < 0.05 compared with model group).

Anxiety-like behaviors of animal are determined by EPMT and OFT. In EPMT, after CFA injection, the number of entries in open arms and retention times of open arms significantly decreased. Moreover, compared with the model group, the number of entries in open arms and retention times of open arms in the GAS-treated group were increased (Figures 4A,B). In the OFT, compared with the blank group, the time spent in the central area decreased in the model group, while the GAS (100 and 200 mg/kg) reversed the reduction caused by CFA (Figure 5). The results show that GAS attenuated CFA-induced anxiety-like behavior.

### Effects of GAS on Ferroptosis-Related Gene Expression

**Figure 6:**
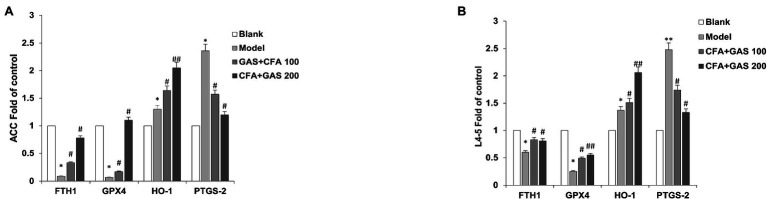
2^–(∆∆Ct)^ value of genes in ACC and L4-5. **(A)** 2^–(∆∆Ct)^ value of ferroptosis-related genes in ACC **(B)** 2^–(∆∆Ct)^ value value of ferroptosis-related genes in L4-5. ^*^p < 0.05 and ^**^p < 0.01 compared with blank group; ^##^*p* < 0.01 and ^#^*p* < 0.05 compared with model group.

The mRNA expressions of FTHI, GPX4, HO-1, and PTGS2 in the anterior cingulate cortex (ACC) and L4-5 of mice on day 14 after CFA injection were detected through qPCR. The relative expression levels of ferroptosis-related genes were shown in Figure 6. In the ACC and L4-5, both FTH1 and GPX4 were significantly decreased on the model group as compared with blank group. Meanwhile, we found that CFA elevated the expression levels of PTGS2 and HO-1. Compared with the model group, FTH1, GPX4, and HO-1 in GAS groups were significantly increased while PTGS2 decreased in a dose-dependent pattern. Taken together, GAS increased the FTHI, GPX4, HO-1, and PTGS2 mRNA expressions but did not change the jejunal microbiota.

### Effects of GAS on Jejunal Structure and Microbiota

**Figure 7:**
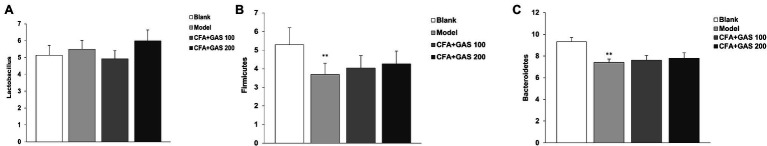
Effect of GAS on jejunal microbiota. **(A)** Effect of GAS on Lactobacillus species. **(B)** Effect of GAS on Firmicutes species. **(C)** Effect of GAS on Bacteroidetes species. ^**^p < 0.01 compared with blank group.

**Figure 8:**
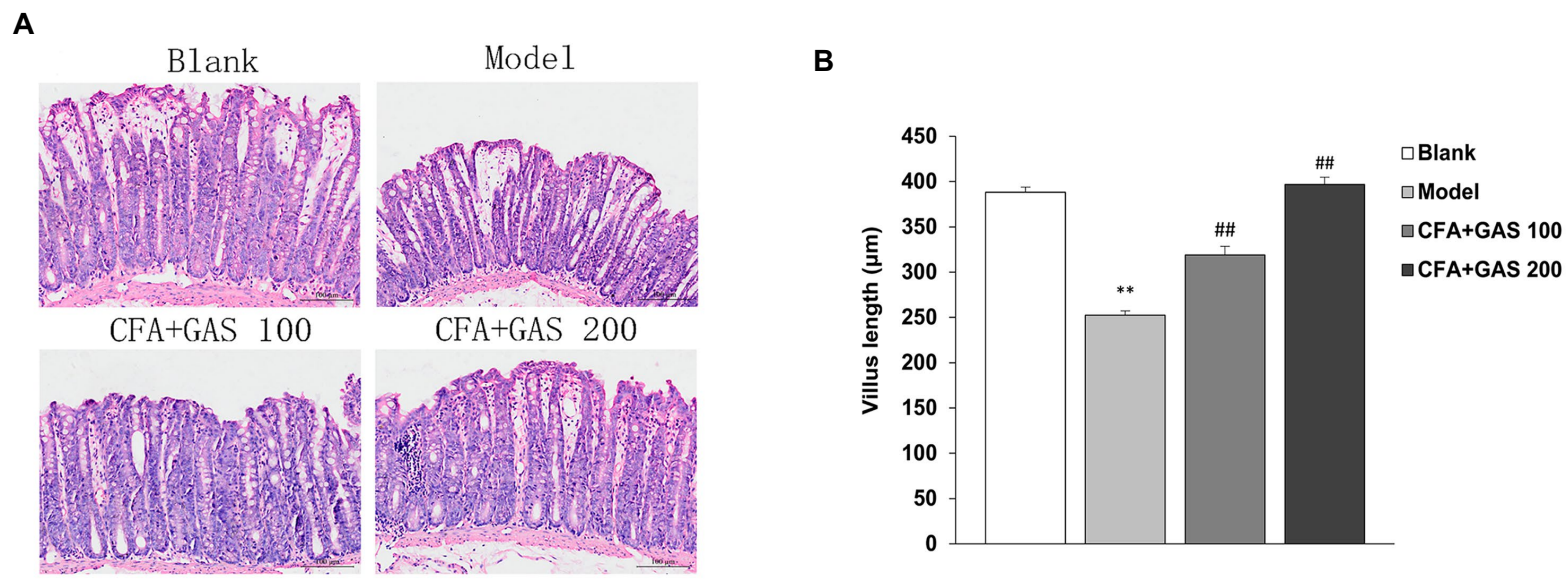
Effect of GAS on jejunal microbiota. **(A)** Characteristics of jejunal villi of mice in each group. **(B)** Effect of GAS on jejunum villus length. ^**^p < 0.01 compared with blank group; ^##^*p* < 0.01 compared with model group.

The effects of GAS jejunal morphological characteristics and microbiota are shown in Figures 7, 8, respectively. The jejunum villus length was decreased in the model group, whereas the villus length was increased in GAS groups (100 and 200 mg/kg). In addition, CFA decreased the Bacteroidetes and Firmicutes species without affecting *Lactobacillus* species. However, the jejunal microbiota did not change after GAS treatment.

## Discussion

Acute inflammatory pain induced by injection of CFA. In this process, rats were allergic to mechanical allodynia and thermal hyperalgesia, and the pain-induced anxiogenic effect lasted for more than 14 days (Nagakura et al., 2003). Clinically, it has been reported that chronic pain leads to mental problems such as anxiety and depression, which seriously reduces the quality of life of patients and hinders their normal life (Gallagher et al., 1995). GAS is a phenolic glucoside with significant analgesic and anti-inflammatory effects. In the CFA-induced chronic inflammatory pain model, we found that mechanical and thermal pain threshold were increased with treatment of GAS in a dose-dependent pattern. In addition, the number of entries in open arms and retention times of open arms were increased by GAS. These studies further confirmed that GAS has powerful analgesic, anti-inflammatory, and anti-anxiety effects in the chronic inflammatory pain model of mice. GAS exerted analgesic and anti-inflammatory effects by decreasing the activation of astrocyte and microglia and the induction of TNF-α and IL-6 in the ACC (Sun et al., 2016). In a mouse model of chemotherapeutic agent-induced neuropathic pain, 5-HT 1A receptor can mediate the powerful antinociceptive of GAS (Guo et al., 2013).

Inflammatory disease (ID) is a series of diseases characterized by inflammatory response, and ferroptosis is closely related to inflammatory response (Andersen et al., 2020). There are some inflammatory factors related to the metabolism of peroxides and arachidonic acid in ferroptosis tissues (Stockwell et al., 2020). Studies have shown that both ferroptosis and inflammatory diseases have the depletion of Gx4 and GSH, the increase of lipid peroxidation products, and the interruption of iron metabolism (Mao et al., 2020). At present, although a variety of molecular mechanisms and signaling pathways can lead to ferroptosis, iron metabolism and lipid peroxidation signaling are the main way to regulate ferroptosis (Dixon et al., 2015). During iron metabolism, excessive iron leads to ferroptosis by producing ROS. Ferritin heavy chain 1 (FTH1), as an iron storage protein complex, is involved in the uptake of excessive iron (Xie et al., 2016). We found that GAS increased the expression of FTH1 and thus balanced intracellular iron levels. The heme oxygenase-1 (HO-1), a major intracellular source of iron (Kwon et al., 2015), plays an important role in ferroptosis and inflammation. It was reported that p38 MAPK phosphorylation could mediate the protective effect of GAS on H_2_O_2_-induced oxidative stress (Zhang et al., 2018). GAS could ameliorate MPP+-induced oxidative stress by regulating the expression of HO-1 in human dopaminergic cells (Jiang et al., 2014). We also demonstrated that GAS increases HO-1 expression, which accelerates the decomposition of heme and inhibits inflammation. In addition, the expressions of glutathione peroxidase4 (GPX4) and prostaglandin-endoperoxide synthase2 (PTGS2) are also important for the induction of ferroptosis.

In our experiments, GAS significantly upregulates the expression of FTH1 and GPX4, decreases PTGS2 expression, and suggests that GAS against ferroptosis by reducing lipid peroxidation. CFA-induced chronic inflammatory pain is accompanied by the ferroptosis of neuronal cells, and GAS has an inhibitory effect on ferroptosis, which is one of the possible mechanisms to protect neuronal cells.

## Data Availability Statement

The original contributions presented in the study are included in the article/supplementary material, further inquiries can be directed to the corresponding author.

## Ethics Statement

All experimental procedures were approved by the Animal Ethics Committee of Southwest Jiaotong University and were conducted in accordance with the university’s animal experiment guidelines.

## Author Contributions

JW and ZH: data collection. XW and XL: conceive and design the study. JW and XL: statistical analysis. XC, JW, and ZH: drafting the manuscript. XC and HL: critical revision of the manuscript. All authors contributed to the article and approved the submitted version.

## Funding

This work was supported by the Science and Technology Department of Sichuan Province (project no.: 2018FZ0096) and the Fundamental Research Funds for the Central Universities (project no.: A0920502052001-10).

## Conflict of Interest

The authors declare that the research was conducted in the absence of any commercial or financial relationships that could be construed as a potential conflict of interest.

## Publisher’s Note

All claims expressed in this article are solely those of the authors and do not necessarily represent those of their affiliated organizations, or those of the publisher, the editors and the reviewers. Any product that may be evaluated in this article, or claim that may be made by its manufacturer, is not guaranteed or endorsed by the publisher.

## Abbreviations

GAS: Gastrodin
CFA: Complete Freund’s adjuvant
EPMT: Elevated plus-maze test
OFT: Open-field test
GPX4: Glutathione peroxidase
FTH1: Ferritin heavy chain 1
HO-1: The heme oxygenase-1
PTGS2: Prostaglandin-endoperoxide synthase2
ROS: Reactive oxygen species
ACC: Anterior cingulate cortex
Nrf2: Nuclear factor erythroid 2-relatedfactor2
